# Development of a Moving Baseline RTK/Motion Sensor-Integrated Positioning-Based Autonomous Driving Algorithm for a Speed Sprayer

**DOI:** 10.3390/s22249881

**Published:** 2022-12-15

**Authors:** Joong-hee Han, Chi-ho Park, Young Yoon Jang

**Affiliations:** 1 Division of Electronics & Information System, DGIST, Daegu Metropolitan City 42988, Republic of Korea; 2 Sungboo IND Ltd., Chilgok-gun 39909, Republic of Korea

**Keywords:** autonomous driving-based spraying work, speed sprayer, sensor fusion, moving baseline real-time kinematic, motion sensor

## Abstract

To address problems such as pesticide poisoning and accidents during pest control work and to enable efficient work in this area, the development of a competitively prices speed sprayer with autonomous driving is required. Accordingly, in order to contribute to developing the commercialization of a low-cost autonomous driving speed sprayer, we developed a positioning algorithm and an autonomous driving-based spraying algorithm by using two low-cost global navigation satellite system (GNSS) modules and a low-cost motion sensor. In order to provide stable navigation solutions from the autonomous driving hardware despite disturbances from the electromagnetic field generated by the spraying device, the proposed positioning algorithm, a moving baseline (MB) real-time kinematic (RTK)/motion sensor-integrated positioning algorithm, was developed using a loosely coupled extended Kalman filter. To compare the yaw estimation performance provided by the MB RTK positioning technique, yaw was calculated by post-processing with two types of positioning algorithms: the MB RTK/motion sensor-integrated positioning algorithm and the GNSS RTK/motion sensor-integrated positioning algorithm. In the static test, the precision of the yaw provided by the MB RTK/motion sensor-integrated positioning algorithm was 0.14°, but with the GNSS RTK/motion sensor-integrated positioning algorithm, the precision of the yaw was 4.53°. The static test results confirmed that the proposed positioning algorithm using the yaw provided by the MB RTK positioning technique based on two GNSS modules for measurement, precisely estimated the yaw even when the spray engine was operating. To perform autonomous driving and spraying, an autonomous driving-based spraying algorithm was developed using the MB RTK/motion sensor-integrated positioning algorithm. As a result of two performance tests based on the proposed algorithm in an orchard, autonomous driving and spraying were stably performed according to the set autonomous driving route and spraying method, and the root mean square (RMS) of the path-following error was 0.06 m.

## 1. Introduction

Various autonomous driving agricultural machines are being developed today, which fuse fourth industrial revolution technologies such as robots, intelligent sensors, and IoT (Internet of Things), with agricultural machines, and are attracting attention as potential solutions to current problems in the agricultural field to improve productivity. Expanding the use of autonomous driving technology-based smart agricultural machinery is expected to address the problem of aging workers, reduce working hours, and improve convenience and efficient cultivation in large-scale farmland. To improve productivity, pest control work is also essential in various farming management operations [1]. In outdoor fruit orchards, pest control accounts for about 30% of orchard management work, and is performed with a speed sprayer, a pesticide-spraying agricultural machine, 8 to 15 times a year [2]. The use of a speed sprayer typically results in a variety of problems, including pesticide poisoning damage caused by spraying pesticides harmful to the human body and safety accidents, such as overturned vehicles. To avoid these problems and perform efficient pest control, the development of a speed sprayer capable of autonomous driving is needed.

GUSS (global unmanned spray system) automation was developed as the world’s first commercialized autonomous orchard sprayer (commonly known as “GUSS”), which uses GNSS (global navigation satellite system) software and laser technology to navigate the canopies of fruit trees from below [3]. In GUSS [4], autonomous driving-based spraying is conducted by using two GNSSs and a LiDAR (light detection and ranging) and could provide live video monitoring through a front-mounted camera. Similar to the commercialized GUSS system, research on an autonomous driving speed sprayer is currently being carried out in order to supplement the shortcomings of sensors, such as GNSS and LiDAR, with the goal of combining multi-sensors for safe and accurate autonomous driving. Cantelli et al. [5] developed a spraying robot, and the absolute position and attitude for autonomous driving of the spraying robot were measured using a high-precision GNSS receiver, motor encoders, and an AHRS (attitude and heading reference system) and a laser scanner was installed for obstacle detection. In [5], it was shown that the maximum cross-track error of autonomous driving was 0.23 m and the preliminary performance of the spraying system during autonomous driving was also evaluated. However, since an expensive GNSS receiver was used for autonomous driving operations, there are limitations in terms of developing and commercializing autonomous driving speed sprayers. Wang et al. [6] studied the design and development of an orchard autonomous navigation system which used a GNSS, a LiDAR, an IMU (inertial measurement unit), and ultrasonic sensors. In [6], it was shown that the average navigation error on a straight section of 10 m was less than 0.15 m and the coverage rate of the leaves of the canopy was about 50% in spraying tests. However, a limitation of the study is that the performance of the autonomous driving of the developed system in the orchard passage was not evaluated, since the autonomous driving test was conducted only on a short straight section of the road. In addition, as in previous studies, applying LiDAR for navigation system operation has advantages in autonomous driving operation as it enables obstacle avoidance and map generation but also raises the development price of the autonomous driving speed sprayer, which may reduce sale competitiveness when commercialized.

The GNSS signal reception environment in the orchard passage where the agricultural machinery is operated has improved due to the recent mechanization of orchard agriculture, leading to changes in the shape of the fruit trees. In addition, because of the availability of various GNSS satellites and the development of multi-GNSS positioning technology, it is possible to determine a precise position in a tree environment, even using a low-cost GNSS module. Advances in MEMS (microelectromechanical system) sensor technology have led to improvements in the performance of low-cost IMU and motion sensors. The development of autonomous driving techniques using low-cost GNSS and auxiliary sensors, such as IMU and motion sensors, is still an attractive approach from a commercial point of view. For this reason, Han et al. [7] developed an autonomous driving technique with a GNSS and a motion sensor-integrated positioning technique. In addition, Han et al. [8] developed a prototype of the autonomous driving hardware for a speed sprayer that costs less than 1000 USD, consisting of a GNSS module, a motion sensor, an embedded board, and an LTE module, and the performance test of the autonomous driving was conducted in an apple farm. In [8], the RMS (root mean square) of the path-following error was 0.1 m, which demonstrated that stable autonomous driving operations were possible in an orchard environment using low-cost sensors. However, a limitation is the inability to present an autonomous driving-based spraying technique for an autonomous driving speed sprayer.

In this study, a modified positioning technique and an autonomous driving-based spraying technique using two low-cost GNSS modules and a low-cost motion sensor were presented by supplementing the limitations of the algorithm presented in [7,8] to help the development of a low-cost autonomous driving speed sprayer. In [7,8], it was shown that the previously proposed positioning technique, which combined a GNSS and a motion sensor, was not able to perform autonomous driving-based spraying because the yaw changed, even in a stationary state, due to the generation of an electromagnetic field caused by the operation of the spraying engine. Due to this problem, the previously presented technique in [7,8] is limited, in that it cannot stably operate autonomous driving-based spraying. Accordingly, this study developed the moving baseline real-time kinematic (MB RTK)/motion sensor-integrated positioning algorithm with MB RTK technology using two GNSS modules in order to minimize the effect of electromagnetic fields during the operation of the spraying engine and to calculate a stable yaw. Moreover, it was analyzed whether the positioning algorithm proposed in this paper calculates yaw more stably compared to the previous positioning algorithm in the previous papers [7,8]. In addition, for the development of the autonomous driving speed sprayer, an autonomous driving-based spraying algorithm using the MB RTK/motion sensor-integrated positioning algorithm not mentioned in the previous papers [7,8] was proposed. Finally, an autonomous driving-based spraying test was conducted in an orchard environment and the autonomous driving performance was evaluated.

The descriptions of the autonomous driving-based spraying technique, including the overall architecture of the speed sprayer, the design of the autonomous driving system, the positioning algorithm, and the autonomous driving-based spraying algorithm are presented in Section 2. The prototype of the autonomous driving speed sprayer is introduced in Section 3. The performance evaluation of the autonomous driving-based spraying algorithm with the MB RTK/motion sensor-integrated positioning algorithm is provided in Section 4. A discussion with comparisons to previous research results can be found in Section 5. Conclusions and future works are given in Section 6.

## 2. Materials and Methods

### 2.1. Overall Architectural Design of the Autonomous Driving-Based Spraying Work System

**Figure 1:**
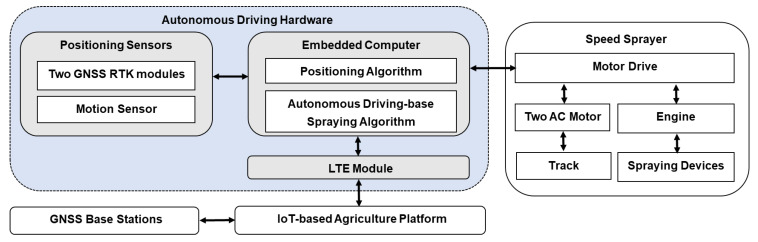
Overall architectural design of an autonomous driving-based spraying work system.

The overall architectural design of the autonomous driving-based speed sprayer system consists of five parts: autonomous driving hardware, a speed sprayer, an IoT-based agriculture platform and GNSS base stations, as shown in Figure 1. The autonomous driving hardware part is composed of positioning sensors, an embedded computer and an LTE (long-term evolution) module. Positioning sensors are included in the two GNSS modules and a motion sensor, which are used to calculate real-time navigation information, such as the position, velocity and attitude of the speed sprayer. The embedded computer is loaded with core algorithms required to operate the autonomous driving-based sprayer, including a positioning algorithm and an autonomous driving-based spraying algorithm. The role of the positioning algorithm is to calculate and provide navigation information using positioning sensor data in real-time. The autonomous driving-based spraying algorithm calculates the vehicle driving and working control parameters in order to operate the autonomous driving-based spraying. The details of the positioning algorithm and the autonomous driving-based spraying algorithm are given in Section 2.2 and Section 2.3, respectively.

The speed sprayer can operate autonomous driving and spraying by receiving control parameters via the autonomous driving hardware. When the control parameters including the rpm of the left and right tracks, engine rpm, spraying direction (left and/or right), and whether to use the fan are received by the motor drive in the speed sprayer, the AC (alternating current) motor and engine operate the driving and spraying, respectively. In addition, information concerning the driving and spraying operation and sensors can be transmitted from the motor drive to the embedded computer to verify their statuses in real-time. To operate the RTK, the GNSS base stations comprise an antenna, a receiver and an internet communication module. These provide RTK correction signals to the GNSS RTK modules in the autonomous driving hardware via an IoT-based agricultural platform. The IoT-based agriculture platform is used to send commands related to the functional operations of the speed sprayer, manage the GNSS base stations and autonomous driving hardware, broadcast the RTK corrections, and collect and provide monitoring information from the speed sprayer.

### 2.2. MB RTK/Motion Sensor-Integrated Positioning Algorithm

**Figure 2:**
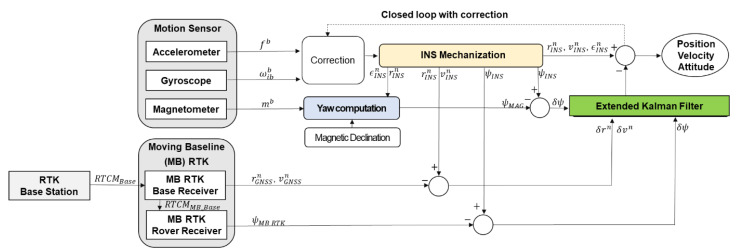
A block diagram of the MB RTK/motion sensor-integrated positioning algorithm. Note: $f^{b}$ is the specific force vector in the body frame, $m/s^{2}$; $\omega_{ib}^{b}$ is the angular velocity vector in the body frame, rad/s; $m^{b}$ is the sign and the magnitude of the earth’s magnetic field vector in the body frame, mG; $r_{GNSS}^{n}$ is the position vector consisting of latitude in units of rad, longitude in units of rad, and ellipsoidal height in units of m calculated from the MB RTK base receiver; $v_{GNSS}^{n}$ is the velocity vector consisting of north, east and down velocity calculated from the MB RTK base receiver, m/s; $\psi_{MB\;RTK}$ is the yaw calculated from the MB RTK rover receiver, rad; $r_{INS}^{n}$ is the position vector consisting of latitude in units of rad, longitude in units of rad, and ellipsoidal height in units of m calculated from the INS mechanization; $v_{INS}^{n}$ is the velocity vector consisting of north, east and down velocity calculated from the INS mechanization, m/s; $\varepsilon_{INS}^{n}$ is the attitude vector consisting of roll, pitch and yaw calculated from the INS mechanization, rad; $\psi_{MAG}$ is the yaw calculated using the magnetometers’ measurement, rad; $\psi_{INS}$ is the yaw calculated from the INS mechanization, rad; $\delta r^{n}$ is the position error vector consisting of latitude in units of rad, longitude in units of rad, and ellipsoidal height in units of m; $\delta v^{n}$ is the velocity error vector consisting of north, east and down velocity, m/s; δψ is the yaw error, rad.

In order to perform stable autonomous driving and spraying work, it is necessary to determine the accurate and continuous position and the yaw of the speed sprayer at a high update rate in real-time. We propose a positioning algorithm based on two GNSS receivers and antennas, and a motion sensor containing a three-axis accelerometer, a three-axis gyroscope and a three-axis magnetometer. A block diagram of the MB RTK/motion sensor-integrated positioning algorithm is shown in Figure 2.

In this study, the MB RTK/motion sensor-integrated positioning algorithm was implemented with a loosely coupled mode using an extended Kalman filter (EKF). A fifteen-state EKF was designed for the proposed algorithm, where the state vector is composed of a navigation part and a sensor part. The nine-error state vector of the navigation part is composed of position errors ($\delta r^{n}$) expressed in the world geodetic system 1984 (WGS84), velocity errors ($\delta v^{n}$) towards the north–east–down (NED) navigation frame and attitude errors ($\delta \varepsilon^{n}$). The six-error state vector of the sensor part consists of accelerometer bias ($\delta b_{a}$) and gyro bias ($\delta b_{g}$). The accelerometer bias and gyro bias are defined by the first-order Gauss–Markov processes. The dynamic model of the proposed algorithm is given in

(1) $$\;\underset{=\delta \dot{x}}{\underbrace{\left[\begin{array}{c} \delta {\dot{r}}^{n}\\ \delta {\dot{v}}^{n}\\ \delta {\dot{\varepsilon }}^{n}\\ \delta {\dot{b}}_{a}\\ \delta {\dot{b}}_{g} \end{array}\right]}=}\underset{=F}{\underbrace{\left[\begin{array}{ccccc} F_{rr} & F_{rv} & F_{r\varepsilon } & 0_{3\times 3} & 0_{3\times 3}\\ F_{vr} & F_{vv} & F_{v\varepsilon } & C_{b}^{n} & 0_{3\times 3}\\ F_{\varepsilon r} & F_{\varepsilon v} & F_{\varepsilon \varepsilon } & 0_{3\times 3} & C_{b}^{n}\\ 0_{3\times 3} & 0_{3\times 3} & 0_{3\times 3} & \beta_{ba} & 0_{3\times 3}\\ 0_{3\times 3} & 0_{3\times 3} & 0_{3\times 3} & 0_{3\times 3} & \beta_{ga} \end{array}\right]}}\underset{=\delta x}{\underbrace{\left[\begin{array}{c} \delta r^{n}\\ \delta v^{n}\\ \delta \varepsilon^{n}\\ \delta b_{a}\\ \delta b_{g} \end{array}\right]}}+\underset{=G}{\underbrace{\left[\begin{array}{cccc} 0_{3\times 3} & 0_{3\times 3} & 0_{3\times 3} & 0_{3\times 3}\\ C_{b}^{n} & 0_{3\times 3} & 0_{3\times 3} & 0_{3\times 3}\\ 0_{3\times 3} & -C_{b}^{n} & 0_{3\times 3} & 0_{3\times 3}\\ 0_{3\times 3} & 0_{3\times 3} & I_{3\times 3} & 0_{3\times 3}\\ 0_{3\times 3} & 0_{3\times 3} & 0_{3\times 3} & I_{3\times 3} \end{array}\right]}}\underset{=\mu }{\underbrace{\left[\begin{array}{c} w_{a}\\ w_{g}\\ w_{ba}\\ w_{bg} \end{array}\right]}},\;$$

where $F_{rr}$, $F_{rv}$, $F_{r\varepsilon }$, $F_{vr}$, $F_{vv}$, $F_{v\varepsilon }$, $F_{\varepsilon r}$, $F_{\varepsilon v}$, and $F_{\varepsilon \varepsilon }$ are system dynamic matrices, which represent the relationship between the position (r), velocity (v) and attitude (ε) state errors; $C_{b}^{n}$ is the transformation matrix from body frame to navigation frame; $0_{n\times m}$ is *n* × *m* zero matrix; $\beta_{ba}$ and $\beta_{ga}$ are the time constant reciprocals of the first-order Gauss–Markov process model for the accelerometer and gyroscope biases, respectively; δx is the error state vector; $\delta r^{n}$ is the error state vector of the position part consisting of latitude in units of rad, longitude in units of rad, and ellipsoidal height in units of m; $\delta v^{n}$ is the error state vector of the velocity part consisting of north, east and down velocity, m/s; $\delta \varepsilon^{n}$ is the error state vector of the attitude part consisting of roll, pitch and yaw, rad; $\delta b_{a}$ is the error state vector of accelerometer bias in the body frame, $m/s^{2}$; $\delta b_{g}$ is the error state vector of gyro bias in the body frame, rad/s; G is the shaping matrix; $I_{3\times 3}$ is the 3 × 3 identity matrix; μ is the white noise vector; $w_{a}$ is the white noises vector for the accelerometers, $m/s^{2}$; $w_{b}$ is the white noise vector for the gyroscopes, rad/s; $w_{ba}$ is the driving noise vector for the accelerometer biases, $m/s^{2}$; and $w_{bg}$ is the driving noise vector for the gyroscope biases, rad/s. The full derivation and definition of the dynamic model’s elements can be found in [9,10].

The measurement model in EKF is generally written as

(2) z=Hδx+w

where *z* is the measurement vector, *H* is the design matrix, *δx* is the error state vector, and *w* is the measurement noise vector.

In this study, the measurement model was considered for the following three situations.

- Magnetometers’ available measurements;
- MB RTK base receiver’s available measurements;
- MB RTK rover receiver’s available measurements.

When the magnetometers in the motion sensor measure the geomagnetic field, the yaw computation and EKF update are sequentially performed. The process of yaw computation using the magnetometers’ measurements is conducted as follows [11]: first, to obtain the horizontal magnetic measurements, the magnetic measurements are converted into measurements in a horizontal plane using a coordinate transformation matrix taken from a sensor frame to a horizontal plane. Then, the ferrous distortion compensation is conducted by using the scale factor and offset of the measurements that had been previously estimated. Once the horizontal magnetic measurements are compensated for by using ferrous distortion, the magnetic yaw is calculated, and then the yaw based on true north is finally calculated through declination angle compensation. The measurement vector for yaw derived from the magnetometer ($z_{MAG}$) is as follows:

(3) $$z_{MAG}=\psi_{INS}-\left(\psi_{MAG}-b_{Yaw}\right)\;$$

where $\psi_{INS}$ is the yaw estimated from the INS mechanization, rad; $\psi_{MAG}$ is the yaw calculated by using the magnetometers’ measurements, rad; and $b_{Yaw}$ is the difference between the yaw calculated by using the magnetometers’ measurements and the yaw calculated from MB RTK, at the previous MB RTK update, rad.

The design matrix for yaw is derived from the magnetometer ($H_{MAG}$) and is expressed as

(4) $$H_{MAG}=\left[\begin{array}{ccccc} 0_{1\times 3} & 0_{1\times 3} & H_{\psi } & 0_{1\times 3} & 0_{1\times 3} \end{array}\right]\;$$

where $0_{1\times 3}$ is the 1 × 3 zero matrix and $H_{\psi }=\left[\begin{array}{ccc} 0 & 0 & 1 \end{array}\right]$ is the design matrix for the attitude part.

When the MB RTK base receiver provides position and velocity, and the measurement vector ($z_{MB\_RTK,\;BASE}$) and the design matrix ($H_{MB\_RTK,\;BASE}$) are as follows:

(5) $$z_{MB\_RTK,\;\;BASE}={\left[\begin{array}{c} r^{n}\\ v^{n} \end{array}\right]}_{INS}-{\left[\begin{array}{c} r^{n}\\ v^{n} \end{array}\right]}_{MB_{RTK},\;BASE}\;$$

(6) $$H_{MB\_RTK,BASE}=\left[\begin{array}{cccccc} I_{3\times 3} & 0_{3\times 3} & 0_{3\times 3} & 0_{3\times 3} & 0_{3\times 3} & 0\\ 0_{3\times 3} & I_{3\times 3} & 0_{3\times 3} & 0_{3\times 3} & 0_{3\times 3} & 0 \end{array}\right]\;$$

where the subscripts INS, MB_RTK, and BASE denote the value estimates from INS mechanization and the data acquisition of the MB RTK base receiver; $r^{n}$ is the position vector consisting of latitude in units of rad, longitude in units of rad, and ellipsoidal height in units of m; $v^{n}$ is the velocity vector consisting of north, east and down velocity, m/s; $I_{3\times 3}$ is the 3 × 3 identity matrix; and$\;0_{3\times 3}$ is the 3 × 3 zero matrix.

When the MB RTK rover receiver provides yaw and the baseline length between base and rover antenna, the difference between the baseline length calculated in MB RTK and the pre-measured baseline length is within a certain length is checked before performing a measurement update. If the difference between the baseline length calculated in the MB RTK rover receiver and the pre-measured baseline length is within a certain length, the measurement vector ($z_{MB\_RTK,\;ROVER}$) and the design matrix ($H_{MB\_RTK,\;ROVER}$) are as follows:

(7) $$z_{MB\_RTK,\;ROVER}=\psi_{INS}-\psi_{MB\_RTK,\;ROVER}$$

(8) $$H_{MB\_RTK,\;\;ROVER}=\left[\begin{array}{cccccc} 0_{1\times 3} & 0_{1\times 3} & H_{\psi } & 0_{1\times 3} & 0_{1\times 3} & 1 \end{array}\right]\;$$

where $\psi_{INS}$ is the yaw estimated from the INS mechanization, rad; $\psi_{MB\_RTK,\;ROVER}$ is the yaw provided by the MB RTK rover receiver, rad; $0_{1\times 3}$ is the 1 × 3 zero matrix; and $H_{\psi }=\left[\begin{array}{ccc} 0 & 0 & 1 \end{array}\right]$ is the design matrix for the attitude part.

After performing the MB RTK yaw measurement update, the difference between the yaw calculated by using the magnetometers’ measurements and the yaw calculated from MB RTK is calculated as:

(9) $$b_{Yaw}=\psi_{MAG}-\psi_{MB\_RTK,\;ROVER}\;$$

where $b_{Yaw}$ is the difference between the yaw calculated by using the magnetometers’ measurements and the yaw calculated from MB RTK, rad; $\psi_{MAG}$ is the yaw calculated by using the magnetometers’ measurement, rad; and $\psi_{MB\_RTK,\;ROVER}$ is the yaw provided by the MB RTK rover receiver, rad.

### 2.3. Autonomous Driving-Based Spaying Algorithm

**Figure 3:**
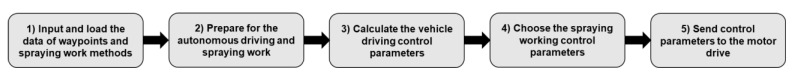
Process of the autonomous driving algorithm.

The autonomous driving-based spraying of the crawler-type speed sprayer requires the rpm of the left and right tracks as the vehicle driving control parameters, the engine rpm, the spraying direction, and whether to use the fan as the working control parameters. Therefore, the role of the autonomous driving-based spaying algorithm is to calculate vehicle driving and working control parameters using the current position and yaw based on the predefined waypoints and the spraying method data assigned to the waypoints. As shown in Figure 3, the process of the autonomous driving-based spraying algorithm is divided into five steps.

The first step is to input and load the data of waypoints and spraying methods for the operation of the autonomous driving-based spraying algorithm. The waypoints data contain information about the autonomous driving route, including waypoint number, geodetic coordinates (latitude, longitude and ellipsoidal height), waypoint type (start point, work point, rotation point and finish point), the azimuth of a straight line created by adjacent waypoints, and angles between straight lines created by adjacent waypoints [8]. The spraying methods data consist of a defined spraying method for each waypoint and include waypoint number, engine rpm, spraying direction (left and/or right), and whether to use the fan or not. 

The second step is to prepare for the autonomous driving and the spraying before operating the autonomous driving-based sprayer. The preparation for autonomous driving begins by aligning the starting location to make sure the speed sprayer is near the starting point of the waypoints for safe autonomous driving. The user manually moves the speed sprayer until the distance between the location of the starting point and the location of the current speed sprayer location is within 0.5 m. When the autonomous driving preparation is complete, the engine rpm is raised to a predefined rpm to enable stable spraying. 

When the preparation for the autonomous driving and the spraying is completed, Steps 3 to 5 of the process in the autonomous driving-based spraying algorithm are sequentially carried out using the position and yaw of the speed sprayer provided by the positioning algorithm. Steps 3 to 5 in the autonomous driving-base spraying algorithm will operate until the speed sprayer arrives at the finish point of the waypoint or deviates from the autonomous driving route. 

**Figure 4:**
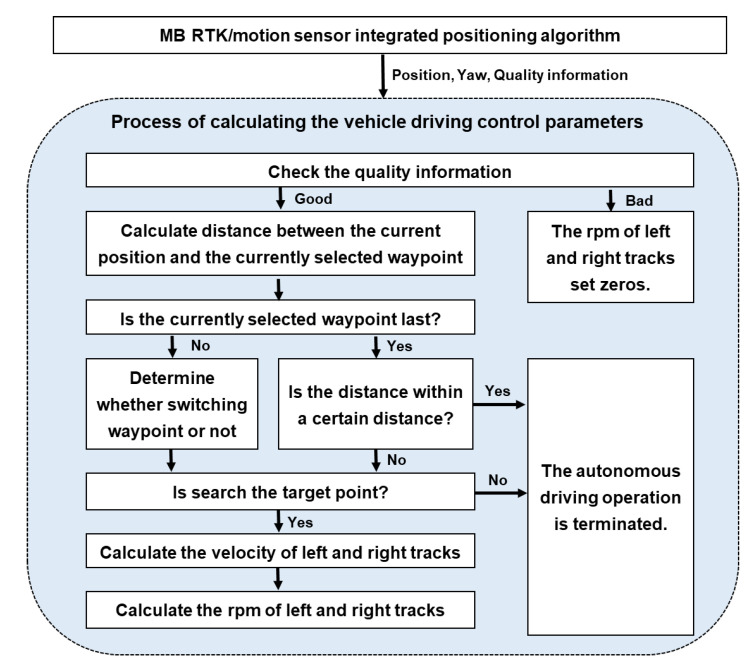
Process of calculating the vehicle driving control parameters, which are the rpm of the left and right tracks.

The third step is to calculate the vehicle driving control parameters (the rpm of the left and right tracks) along the desired vehicle course based on the waypoints and the speed sprayer’s current position and yaw. As shown in Figure 4, the process for calculating the rpm of the left and right tracks as the vehicle driving control parameters are summarized as follows. First, when the MB RTK/motion sensor-integrated positioning algorithm provides position, yaw and quality information (the age of the GNSS measurement update with the resolved ambiguity and the precision of position), it determines whether or not to drive the speed sprayer, using the quality information. The conditions for quality information for autonomous driving are the age of the GNSS measurement update with a resolved ambiguity that does not exceed 2 s and the precision of position being lower than 0.5 m. Next, the distance between the current position and the currently selected waypoint is calculated, and based on this, whether to continue using the current waypoint or to switch to the next waypoint is determined. If the last waypoint is selected and the distance between the current position and the waypoint is within a certain distance, it is regarded as having arrived at the final destination and the autonomous driving operation is terminated.

If the speed sprayer does not arrive at the final destination, a target point, which is a location to be reached at the next epoch, is searched for. To search the target point, this paper applied the enclosed baseline of the sight guidance method [12]. The enclosed baseline of the sight guidance method is used to calculate a target point as a point intersection between the straight line created between the current waypoint and the previous waypoint and within a circle with a radius enclosing the current position. If the target point cannot be calculated, it is regarded as out of the autonomous driving path and the autonomous driving is terminated. If the target point is successfully calculated, the velocity of the left and right tracks needed to reach the target point at the next epoch are calculated using the current position, yaw, and the location of the target point. Finally, the vehicle driving control parameters, the rpm of the left and right tracks, are calculated using a scale that converts the track speed into rpm. In order to calculate the vehicle driving control parameters, a detailed method and formula were described in [7,8].

The fourth step is to choose the spraying method corresponding to the currently selected waypoint number in the data of spraying methods. If there is no spraying method for the currently selected waypoint or the autonomous driving is terminated, then the spraying control parameters (engine rpm, spraying direction and blowing) are generated for regions that have not been sprayed. In the last step, the rpm of the left and right tracks, engine rpm, spraying direction (left and/or right), and whether to use the fan are transmitted to the motor drive. In this way, the speed sprayer performs the spraying operation based on autonomous driving.

## 3. Prototype of Autonomous Driving Speed Sprayer

**Figure 5:**
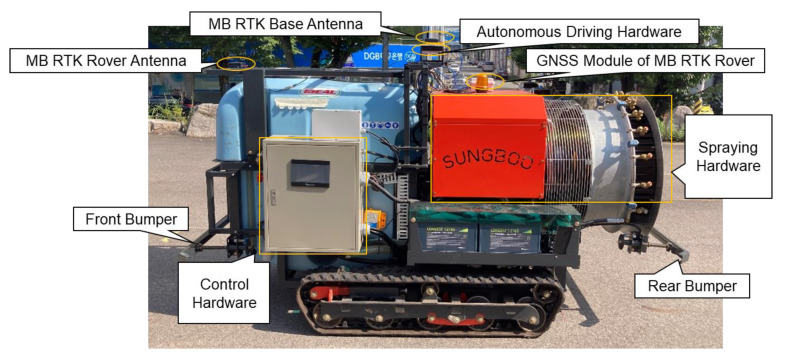
Prototype autonomous driving speed sprayer.

The prototype of an autonomous driving speed sprayer developed by the Sungboo Industry Company is shown in Figure 5. This prototype includes a crawler-type driving system for smooth driving and efficient control in orchard terrain, and designed and manufactured driving and spraying devices. For the driving device, two DC (direct current) 48 V, 2.0 kW AC motors were independently applied to both sides of the tracks. The motor control drive is configured to separately operate the two motor drivers separately as master and slave, and the main controller is configured to control the motor drive, various outputs and spraying. A 500 L tank was utilized as the chemical container, and the spraying hardware was manufactured with a basic speed sprayer configuration, with a spray device and a nozzle. A diesel engine was additionally mounted for spraying the chemical liquid, and was manufactured so that the driving battery is able to be charged through an alternator. Bumper devices were installed in the front and rear of the speed sprayer for any emergency stop caused by obstacle collisions during autonomous driving. The external dimensions of the speed sprayer are 2.183 m (length) × 1.300 m (width). The autonomous driving hardware is mounted on the top of the middle of the spray sprayer. The MB RTK base antenna is mounted on the top of the autonomous driving hardware, and the MB RTK rover antenna is installed on the top front of the speed sprayer. The baseline length between the MB RTK base antenna and the MB RTK rover antenna is about 1.2 m.

**Figure 6:**
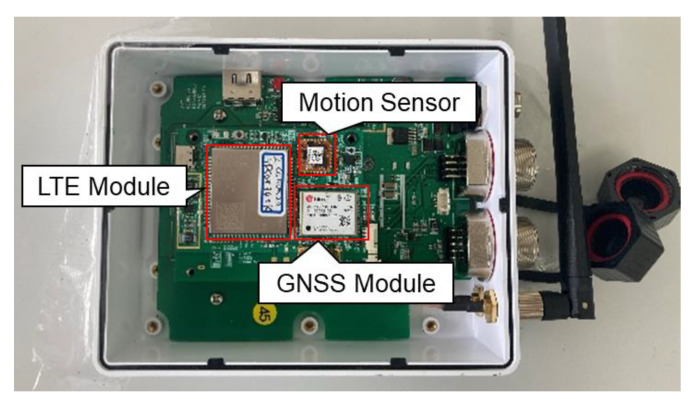
Autonomous driving hardware.

The autonomous driving hardware uses a multi-sensor fusion-positioning device manufactured by the H&I company, as shown in Figure 6. The autonomous driving hardware includes a GNSS module (u-blox’s ZED-F9P), a motion sensor (Xsens’s MTi-1), an embedded computer (raspberry pi 4 model), and an LTE module. The u-blox ZED-F9P is a multi-band GNSS RTK module that provides positions with centimeter-level accuracy using GPS/QZSS (Quasi-Zenith Satellite System), GLONASS (GLObal Navigation Satellite System), BeiDou, and Galileo and supports the MB RTK technique. The price of the ZED-F9P was about 199 USD. In order to receive Radio Technical Commission for Maritime Services (RTCM) correction data from the RTK base station to operate the GNSS RTK, H&I’s RTK platform was used. Xsens’s MTi-1 contains a three-axis accelerometer, a three-axis gyroscope, and a three-axis magnetometer. The three-axis accelerometer has a measurement range of ±16 g, an in-run bias stability of 0.03 mg, and a noise density of 120 $\mathrm{ug}/\sqrt{Hz}$; the three-axis gyroscope has a measurement range of ±2000 °/s, an in-run bias stability of 10 °/h, and a noise density of 0.007 ${}^{\circ}/s/\sqrt{Hz}$; and the three-axis magnetometer has a measurement range of ±8 G, a total RMS noise of 0.5 mG, and a resolution of 0.25 mG. The price of the MTi-1 was about 190 USD. Since only one GNSS module was installed in the autonomous driving hardware, an additional GNSS module was mounted on the speed sprayer to operate the MB RTK and was connected via a USB (universal serial bus). The GNSS antenna used a Hi-Target’s ATCZ-436 model, which supports GPS, GLONASS, Beidou, Galileo, and other constellation satellite signals. RS (recommended standard)-422 was applied for data communication between the motor drive of the speed sprayer and the autonomous driving hardware.

## 4. Results

### 4.1. Performance Evaluation of Yaw Estimation

A static test was carried out to evaluate the yaw estimation performance of the proposed positioning algorithm. The configurations of the positioning modules used for the test were as follows. The output rate of the motion sensor module, including a three-axis accelerometer, a three-axis gyroscope, and a three-axis magnetometer, was set to 100 Hz. The GNSS constellations for the two GNSS receiver modules consisting of a base and a rover were set for the concurrent reception of GPS, GLONASS, Galileo, Beidou, and QZSS. The position and velocity output rate of the MB RTK base receiver module was set to 5 Hz, and the yaw output rate of the MB RTK rover receiver module was set to 1 Hz. Static test data were obtained over ca. 6 min in a parking lot with a normal GNSS signal reception environment. 

In order to analyze the yaw performance during the operation of the diesel engine and spraying function, the experiment was conducted as follows. The diesel engine for spraying was turned on 1 min after the start of data acquisition and the spraying function was activated 1 min after the spray engine was turned on. After 2 min of the activated spraying function, the diesel engine and the function were turned off, and data were acquired over 2 min.

To compare the yaw estimation performance when yaw was provided by the MB RTK positioning technique, yaw was calculated by post-processing two types of positioning algorithms, the GNSS RTK/motion sensor-integrated positioning algorithm in [7,8] and MB RTK/motion sensor-integrated positioning algorithm in this paper. In the GNSS RTK/motions sensor-integrated positioning algorithm, the measurement update rate of the GNSS RTK-based position and velocity and the update rate of the magnetometer-based yaw was set to 5 Hz and 2 Hz, respectively. The MB RTK/motion sensor-integrated positioning algorithm is different than the GNSS RTK/motion sensor-integrated positioning algorithm, in that it performs the measurement update using the yaw provided by MB RTK at 1 s intervals. The output rate of the two types of positioning algorithms was set to 100 Hz, which was the same as the output rate of the motion sensor module.

**Figure 7:**
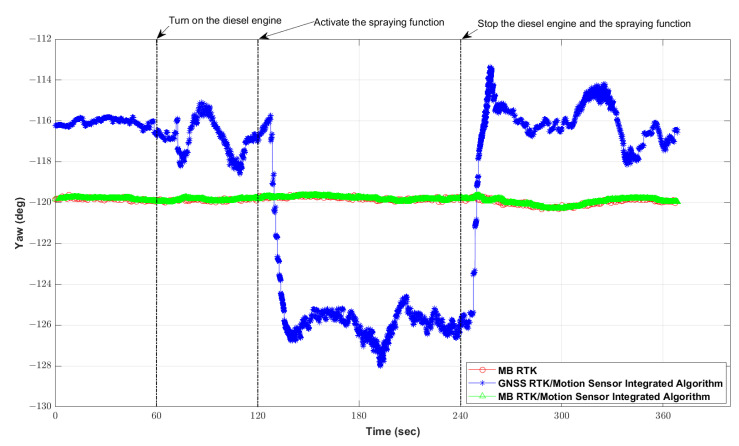
Estimated yaw depending on the type of algorithm.

Figure 7 shows the yaw outputs from the two types of positioning algorithms and MB RTK, and the statistics for the estimated yaw depending on the types of algorithms are summarized in Table 1. As shown in Figure 7, all of the positioning algorithms calculated a constant yaw value before turning on the diesel engine, but after turning on the diesel engine, the yaw value for the GNSS RTK motion sensor-integrated positioning algorithm changed. When the spraying function was active, the yaw value calculated by the GNSS RTK/motion sensor-integrated positioning algorithm changed by up to ca. 10°. After stopping the diesel engine and the spraying function, the yaw value calculated by the GNSS RTK/motion sensor-integrated positioning algorithm appeared to be different from the initial yaw value, even though it was in a static state. The results suggest that the calculated yaw value changed because measurements performed by the magnetometers in the motion sensor were distorted by an electromagnetic field generated during operation of the diesel engine and the spraying function. 

In contrast, with the MB RTK/motion sensor-integrated positioning algorithm, a constant yaw value was calculated regardless of whether the diesel engine or the spraying function were operating or not. Since yaw is calculated by MB RTK using measurements based on signals received from GNSS, it is not affected by electromagnetic fields generated by the diesel engine or the spraying function and can thus provide a stable yaw value. These results confirmed that the proposed MB RTK/motion sensor-integrated positioning algorithm using the yaw provided by MB RTK as a measurement update can estimate the yaw more reliably than the GNSS RTK/motion sensor-integrated positioning algorithm.

### 4.2. Performance Evaluation of Autonomous Driving-Based Spraying Algorithm

In order to evaluate the performance of the proposed autonomous driving-based spraying algorithm with the MB RTK/motion sensor-integrated algorithm, tests were conducted at an apple farm (Young Cheon, Korea) in August 2022. The waypoints for the autonomous driving route were created using a path generation algorithm developed by [13] using location data collected by manually driving the speed sprayer. The minimum distance between adjacent waypoints, which are options for creating waypoints, and the angle between adjacent straight lines that determine the rotation section, were set to 0.5 m and 10°, respectively. The total length of the generated autonomous driving trajectory was 943.31 m, the number of waypoints was 1094, and the range of the distance between adjacent waypoints was 0.06 to 3.49 m. The trajectory consisted of 14 straight sections and 13 rotation sections.

**Figure 8:**
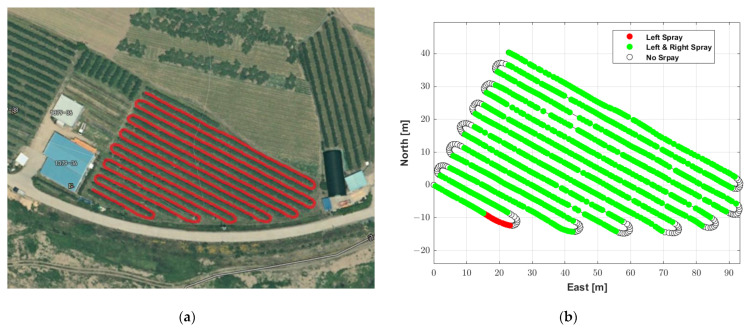
Autonomous driving trajectory used to evaluate the autonomous driving performance. (**a**) Autonomous driving trajectory, (**b**) waypoints with spraying direction.

Figure 8a shows the generated autonomous driving trajectory, shown as a red line overlaid on an aerial photo. Apple trees were planted on the left and right sides of the straight section, and the width is about 3.5 m. The data for the spraying method were manually created by considering the existence of trees around the position of the waypoint and whether a rotation section was present. Figure 8b shows the waypoints of the autonomous driving trajectory and the spraying direction at the waypoints.

The configurations of the algorithm used for the autonomous driving-based spraying test were as follows. The control interval of the autonomous driving-based spraying was set to 0.01 s, equal to the output rate of the MB RTK/motion sensor-integrated positioning algorithm. The maximum and minimum speeds of the autonomous driving were set to 4 km/h and 0.5 km/h, respectively, in consideration of the general working speed of the speed sprayer and stable rotational driving. The range of acceptable radius for the switching waypoints was set to 0.2 to 1 m, and this radius changed depending on the angle between the two straight lines created by two adjacent waypoints relative to the selected waypoint. The maximum radius for searching a target point was set to 2 m. The rpm of the diesel engine for spraying was set to 1800. When the distance between the last waypoint and the location of the speed sprayer was within 0.3 m, the autonomous driving operation was set to finish.

**Figure 9:**
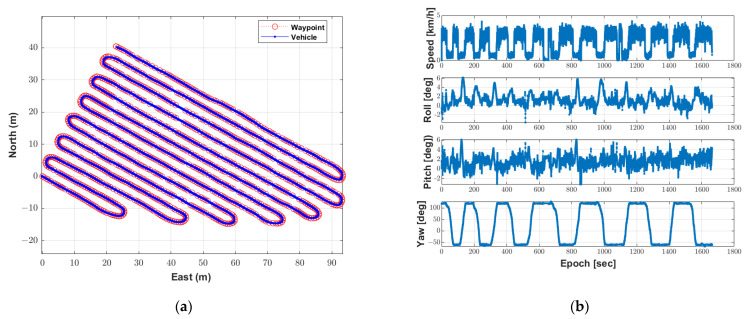
Location, speed and attitude of the speed sprayer during the first test of the autonomous driving-based spraying. (**a**) Location of the speed sprayer with waypoints. (**b**) Speed and velocity of the speed sprayer.

The autonomous driving experiment was conducted twice using the waypoints and the configurations of the algorithm. The first result of the autonomous driving-based spraying test using the autonomous driving trajectory was as follows. Figure 9a shows where the speed sprayer traveled during autonomous driving with waypoints. The driving time was 27 min 43.58 s, and the autonomous driving operation was completed along the waypoints. Figure 9b shows the speed and attitude of the speed sprayer during the autonomous driving-based spraying test. The maximum speed calculated by the MB RTK/motion sensor-integrated positioning algorithm was 4.4 km/h, which was similar to the maximum speed of 4 km/h in the autonomous driving configurations. 

**Figure 10:**
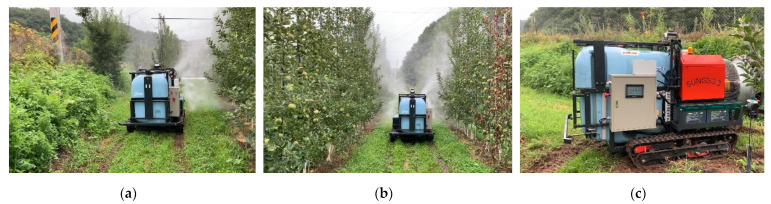
The speed sprayer operating during the first test of the autonomous driving-based spraying. (**a**) Left spraying zone, (**b**) Left and right spraying zone, (**c**) No spraying zone (rotation section).

In addition, it can be seen that deceleration occurred when entering the rotation section and acceleration occurred when entering the straight section. The range of attitude roll and pitch was −3.7° to 6.2° and −3.2° to 6.1°, respectively. As can be seen from the range of the roll and pitch, the autonomous driving operation was completed despite the uneven areas. Figure 10 shows that the autonomous driving-based spraying was performed based on the spraying direction assigned to the waypoint.

**Figure 11:**
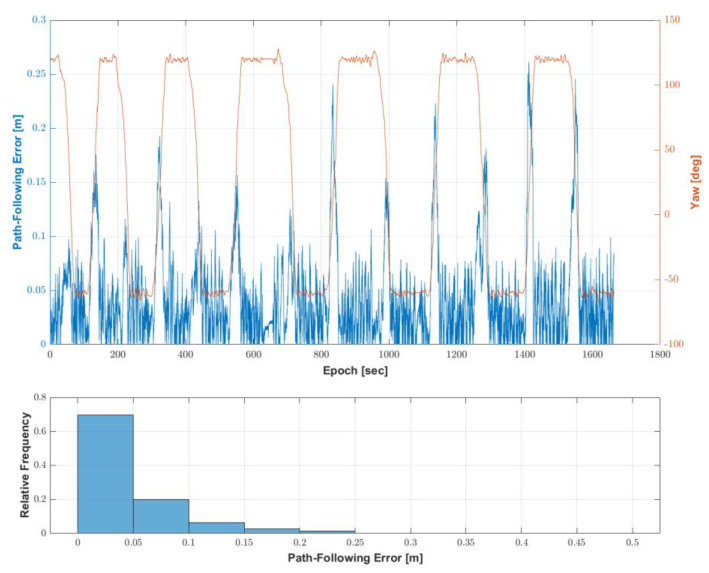
The path-following error during the first test of the autonomous driving-based spraying test.

To evaluate the performance of the autonomous driving, a path-following error was calculated. The path-following error was calculated as the shortest distance between the speed sprayer’s location and a straight line between two consecutive points near the speed sprayer’s location at every epoch [7]. Figure 11 shows the path-following error with the speed sprayer’s yaw at every epoch and the histogram of the path-following error in the first test of the autonomous driving-based spraying. As can be seen in Figure 11, the overall path-following error was less than 0.1 m in the straight section, but the path-following error was larger in the rotation section than in the straight section. This result is considered to be caused by track slip during the speed sprayer rotation. The distribution of the path tracking error was found to be 70% below 0.05 m and 90% below 0.1 m. The maximum error of the path-following was 0.28 m, and the RMS of the path-following error was 0.06 m. 

**Figure 12:**
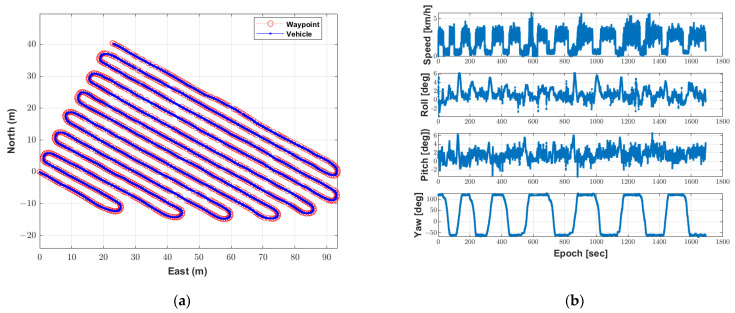
Location, speed and attitude of the speed sprayer during the second test of the autonomous driving-based spraying. (**a**) Location of the speed sprayer with waypoints. (**b**) Speed and velocity of the speed sprayer.

The results of the second test using the same waypoints and settings as the first test were as follows. Figure 12 shows the location, speed and attitude of the speed sprayer during the second test of the autonomous driving-based spraying. The autonomous driving operation was completed along the waypoints. The driving time and the maximum speed were 28 min 11.86 s and 5.8 km/h, respectively.

**Figure 13:**
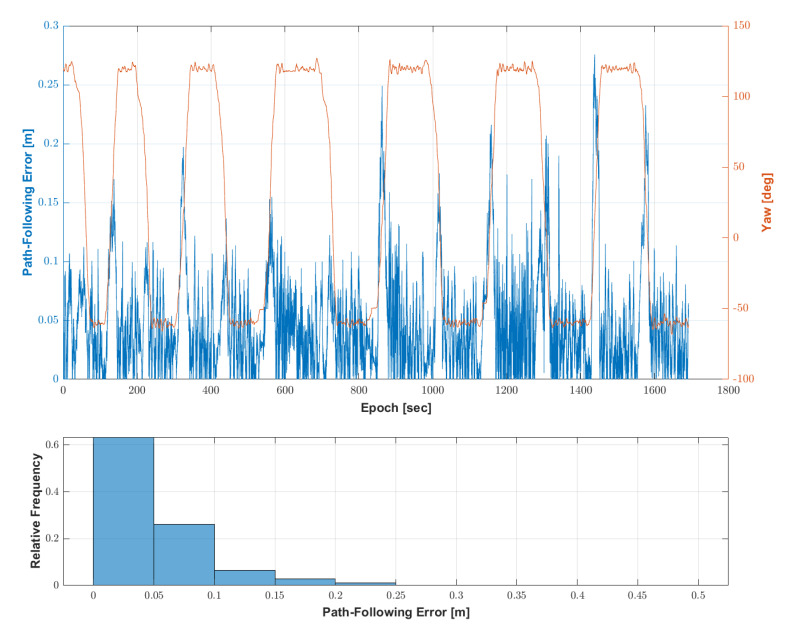
The path-following error during the second test of the autonomous driving-based spraying.

Figure 13 shows the path-following error with the speed sprayer’s yaw at every epoch and the histogram of the path-following error during the second test of the autonomous driving-based spraying. Similar to the result of the first test, the overall path-following error was less than 0.1 m in the straight section, but the path-following error was larger in the rotation section than in the straight section. The distribution of the path tracking error was found to be 70% below 0.05 m and 93% below 0.1 m. The maximum error of the path-following was 0.29 m. As in the first test, the RMS of the path-following error was 0.06 m.

## 5. Discussion

To address the unstableness of yaw calculated by the GNSS RTK/motion sensor-integrated positioning algorithm in [7,8], which is caused by the generation of an electromagnetic field from the spraying engine, this paper proposed the MB RTK/motion sensor-integrated positioning algorithm based on two GNSS modules and a motion sensor. As a result of comparing the yaw estimation performance through the static test, the precision of the yaw provided by the MB RTK/motion sensor-integrated positioning algorithm was 0.14° degrees, but with the GNSS RTK/motion sensor-integrated positioning algorithm, the precision of the yaw was 4.53°. It was found that the MB RTK/motion sensor-integrated positioning algorithm-calculated yaw was more stable than that of the GNSS RTK/motion sensor-integrated positioning algorithm, regardless of whether the diesel engine or the spraying function were operating or not. As a result of the performance evaluation of the proposed autonomous driving-based spraying algorithm with the MB RTK/motion sensor-integrated algorithm, the RMS of the path-following error of two tests was 0.06 m. As the RMS of the path-following error evaluated by the same orchard in [8] was 0.10 m, it was found that the autonomous driving algorithm based on the proposed algorithm in this paper operates more stably. In addition, as the maximum path-following error was 0.29 m, which was similar to the maximum error of 0.23 m in [5], it was demonstrated that stable autonomous driving is possible with low-cost positioning sensors. Therefore, it is considered appropriate to use the proposed positioning algorithm in this paper in order to realize the autonomous driving operation of agricultural machinery that operates the working equipment. Furthermore, since the autonomous driving technique using low-cost positioning sensors can be stably operated through the proposed method, it is considered to be able to contribute to the commercialization of an autonomous driving speed sprayer through the development of low-cost autonomous driving hardware.

## 6. Conclusions and Future Works

In order to design an autonomous driving-based speed sprayer, this study developed a positioning algorithm and an autonomous driving-based spraying algorithm. To provide stable position and yaw control despite the influence of electromagnetic fields that may be generated during the operation of the speed sprayer, the proposed positioning algorithm, the MB RTK/motion sensor-integrated positioning algorithm, was developed using two GNSS modules and a motion sensor. A test was conducted to evaluate whether the MB/RTK/motion sensor-integrated positioning algorithm was able to successfully estimate a stable yaw. The test results confirmed that the proposed positioning algorithm applied to the yaw provided by MB RTK was able to estimate stable yaw without being affected by the electromagnetic field generated by the diesel engine and spraying operation, compared to the algorithm without MB RTK. Next, we developed an autonomous driving-based spraying algorithm based on the proposed positioning algorithm in order to enable spraying during the autonomous driving of the speed sprayer. The testing of the proposed autonomous driving-based spraying algorithm was carried out in an apple farm using a prototype of an autonomous driving speed sprayer. Over an autonomous driving trajectory of about 943 m, the RMS of the path-following error was 0.06 m. These results demonstrate that is possible to develop a commercial autonomous driving speed sprayer using the proposed positioning algorithm and autonomous driving-based spraying algorithm, based on two low-cost GNSS modules and a low-cost motion sensor.

To commercialize the autonomous driving speed sprayer using the proposed algorithms, additional studies will be carried out as follows. Firstly, we will conduct an evaluation of the spraying effectiveness of the proposed autonomous driving-based spraying algorithm, which was not performed in this paper. In addition, we will improve the stability and performance of the proposed algorithms through a number of tests in various orchards utilizing the speed sprayer. Furthermore, we will develop various functions that are able to respond to various situations encountered during autonomous driving, such as a function enabling the return of the sprayer to its starting point when there is insufficient liquid or battery and a function enabling a change in spraying methods during autonomous driving. Secondly, since the prototype of the autonomous driving speed sprayer was only equipped with bumper sensors designed to stop after rather than before a collision, we will develop an obstacle detection function using low-cost sensors, such as ultrasonic sensors and radar. Finally, we will develop an orchard map creation technique that combines positioning sensors, vision sensors and LiDAR to enable the autonomous driving of a variety of agricultural machinery in the orchard.

## Figures and Tables

**Table 1:** Statistics of estimated yaw depending on the type of algorithm (unit: °).

Type	Range	Mean	Standard Deviation
GNSS RTK/Motion Sensor-Integrated Algorithm	−113.34 ~ −128.01	−119.36	4.53
MB RTK/Motion Sensor-Integrated Algorithm	−119.61 ~ −120.30	−119.86	0.14
MB RTK	−119.61 ~ −120.32	−119.88	0.14

## Data Availability

Not applicable.
